# How π back-donation quantitatively controls the CO stretching response in classical and non-classical metal carbonyl complexes†
†Electronic supplementary information (ESI) available: Additional figures and DFT-optimized XYZ geometries for all the complexes studied. See DOI: 10.1039/c5sc02971f


**DOI:** 10.1039/c5sc02971f

**Published:** 2015-10-26

**Authors:** Giovanni Bistoni, Sergio Rampino, Nicola Scafuri, Gianluca Ciancaleoni, Daniele Zuccaccia, Leonardo Belpassi, Francesco Tarantelli

**Affiliations:** a Dipartimento di Chimica , Biologia e Biotecnologie , Università di Perugia , Via Elce di Sotto 8 , 06123 Perugia , Italy; b Istituto di Scienze e Tecnologie Molecolari del CNR , Via Elce di Sotto 8 , 06123 Perugia , Italy . Email: giovanni@thch.unipg.it ; Email: srampino@thch.unipg.it ; Email: francesco.tarantelli@unipg.it; c Institut Charles Gerhardt , Université Moltpellier 2 , ENSCM 5253, cc 1501, Place Eugène Bataillon , 34095 Montpellier Cedex 5 , France; d Dipartimento di Chimica , Fisica e Ambiente , Via del Cotonificio 108 , 33100 Udine , Italy

## Abstract

The CO stretching response upon coordination to a metal M to form [(L)_*n*_M(CO)]^*m*^ complexes (L is an auxiliary ligand) is investigated in relation to the σ donation and π back-donation components of the M–CO bond and to the electrostatic effect exerted by the ligand–metal fragment. Our analysis encompasses over 30 carbonyls, in which the relative importance of donation, back-donation and electrostatics are varied either through the ligand in a series of [(L)Au(CO)]^0/+^ gold(i) complexes, or through the metal in a series of anionic, neutral and cationic homoleptic carbonyls. Charge-displacement analysis is used to obtain well-defined, consistent measures of σ donation and π back-donation charges, as well as to quantify the σ and π components of CO polarization. It is found that all complexes feature a comparable charge flow of σ symmetry (both in the M–CO bonding region and in the CO fragment itself), which is therefore largely uncorrelated to CO response. By contrast, π back-donation is exceptionally variable and is found to correlate tightly with the change in CO bond distance, with the shift in CO stretching frequency, and with the extent and direction (C → O or C ← O) of the CO π polarization. As a result, we conclusively show that π back-donation can be an important bond component also in non-classical carbonyls and we provide the framework in which the spectroscopic data on coordinated CO can be used to extract quantitative information on the π donor properties of metal–ligand moieties.

## Abstract 2

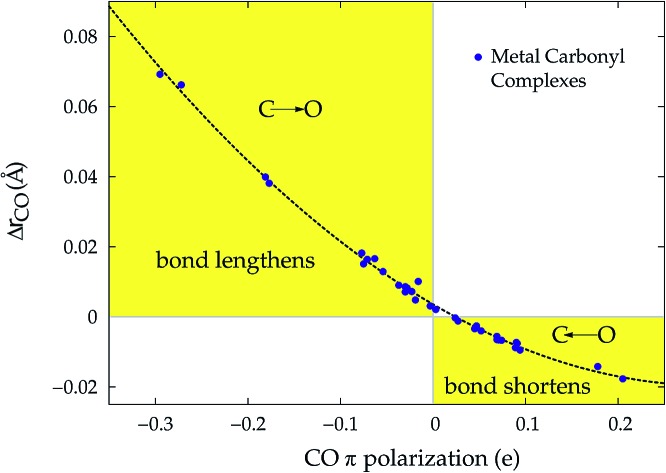
We definitively show that the CO stretching response to metal coordination is driven exclusively by π polarization, which quantitatively correlates with π back-donation and changes in CO bond length and frequency.

## 1 Introduction

The high affinity of carbon monoxide (CO) towards metals (M) has been known since the end of the nineteenth century^1^ and its relevance has kept growing thereafter, both in pure^2^,^3^ and applied chemistry.^4^,^5^ This has led many chemists to study in detail the coordination bond between M and CO in metal–carbonyl complexes, which is commonly described in terms of the Dewar–Chatt–Duncanson (DCD) model.^6^–^8^ According to this scheme, the interaction between M and CO involves the donation of electron charge from the carbon's lone pair to the empty M orbitals of σ symmetry (M ← CO σ donation), and a back-donation from filled M to empty CO orbitals of π symmetry (M → CO π back-donation). The effectiveness of this model for the description of the M–CO bond has been consolidated over the years by a large number of theoretical studies based on a variety of techniques, including energy^9^,^10^ and charge^11^ decomposition schemes, Natural Bond Orbitals (NBO) analysis,^12^ Electron Localization Function (ELF) approaches^13^ and the Quantum Theory of Atoms In Molecules (QTAIM).^14^–^17^

On the experimental side, discussions on the nature of the M–CO bond are mostly based on the analysis of the variation in the CO stretching frequency *ν*_CO_ (*via* IR spectroscopy) and bond distance *r*_CO_ (*via* X-ray crystallography) with respect to free CO (*ν*_free-CO_ = 2143 cm^–1^, *r*_free-CO_ = 1.12822 Å). In most metal–carbonyl complexes the CO bond appears weakened, *i.e.*, the stretching frequency decreases (Δ*ν*_CO_ = *ν*_CO_ – *ν*_free-CO_ < 0) and the bond distance increases (Δ*r*_CO_ = *r*_CO_ – *r*_free-CO_ > 0), but in a minority of (mainly late-metal cationic) complexes, which are sometimes termed “non-classical”,^18^ the CO bond appears strengthened (Δ*ν*_CO_ > 0 and Δ*r*_CO_ < 0). These differences in the CO stretching response to the M–CO bond formation in metal carbonyl complexes are commonly explained in terms of the relative importance of the DCD constituents of the M–CO bond. In particular, M → CO π back-donation is represented as exerting a bond-weakening effect on CO, while M ← CO σ donation is thought to act in the opposite way.^19^,^20^ This view relies on a molecular-orbital picture in which both the π acceptor and σ donor CO orbitals have a C–O anti-bonding character. However, while there is general agreement on the effect thus played by π back-donation, the role of σ donation has been brought into question in the last fifteen years.^21^–^23^ In particular, these studies suggest that the σ CO donor orbital has, rather, a weak bonding character and that the CO bond strengthening in non-classical complexes is an electrostatic effect due to the (positively charged) ligand–metal moiety, whereby the CO bonding orbitals of both σ and π symmetry are polarized in the C ← O direction, thus enhancing the covalency of the CO bond.

One way to schematically depict M(CO) bonding resorts to a simple Valence Bond (VB) picture. Focusing on the M(CO) moiety of a generic [(L)_*n*_M(CO)]^*m*^ complex, three VB structures differing for the extent of π back-donation can be written:^(a) –^M–C≡O^+^ ↔ ^(b)^ M=C=O ↔ ^(c) +^M≡C–O^–^

In going from structure (a) to structure (b) and (c), where one has zero, one and two π* orbitals of CO engaged in back-bonding, the CO bond multiplicity goes from three to two to one. The relative weight of each structure will of course depend on the π donor properties of the specific [(L)_*n*_M]^*m*^ fragment. At the same time, the electronic structure of CO is also affected by the electric field generated by this fragment, especially in those cases when *m* ≠ 0. For CO in the presence of an electric field generated, for instance, by a positively charged metal fragment (exemplified here with the symbol ⊕), three analogue VB structures can be written:^(d)^ ⊕ ^–^C≡O^+^ ↔ ^(e)^ ⊕ C=O ↔ ^(f)^ ⊕ ^+^C–O^–^

The presence of such electric field would in this case favour the triple bonded structure (d) over structures (e) and (f) featuring a double and single bond, respectively (an opposite effect, of course, is expected to occur when the electric field is generated by an anionic ligand–metal fragment). The DCD bonding structure and the electrostatic polarization effect may thus *a priori* act in different directions with different weight, so that their interplay in driving CO stretching response may be difficult to disentangle.

**Fig. 1:**
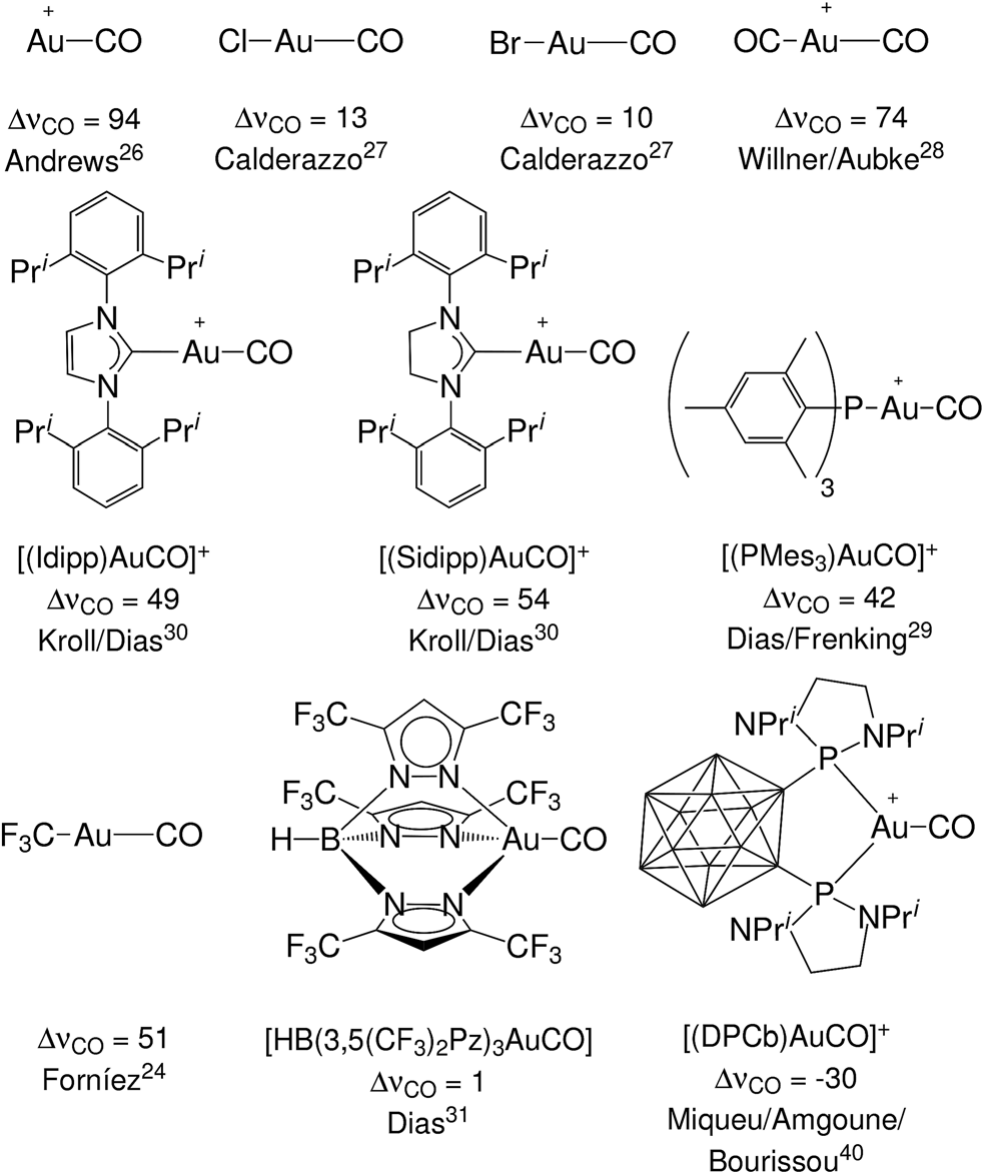
The experimentally characterized gold(i) carbonyl complexes discussed in this work, with the observed carbonyl stretching frequency shifts, Δ*ν*_CO_ (in cm^–1^), and literature references.

Still, however, carbonyl complexes showing blue shifted (Δ*ν*_CO_ > 0) CO stretching frequencies are most often assumed to lack back-donation from the metallic fragment.^18^,^24^,^25^ Exemplary in this respect is the set of complexes [(L)Au(CO)]^0/+^ of gold(i) that have been experimentally characterized.^24^,^26^–^31^ Until last year, to our knowledge, nine gold(i) carbonyl complexes had been spectroscopically characterized: the ligand free [Au(CO)]^+^ (observed in neon matrix^26^) and its derivatives with ligands Cl^–^,^27^ Br^–^,^27^ CF_3_^–^,^24^ CO,^28^ Mes_3_P,^29^ SIdipp,^30^ Idipp^30^ and [HB(3,5-(CF_3_)_2_Pz)_3_]^–^,^31^ where Mes stands for 2,4,6-Me_3_C_6_H_2_, SIdipp for 1,3-bis(2,6-diisopropylphenyl)imidazolin-2-ylidene, Idipp for 1,3-bis(2,6-diisopropylphenyl)imidazol-2-ylidene and [HB(3,5-(CF_3_)_2_Pz)_3_]^–^ is a fluorinated tris(pyrazol)borate ligand. They all exhibit blue shift of the CO frequency and therefore are classified as non classical. This has been taken by some authors as proof that the gold fragment gives poor or no back-donation.^32^,^33^ However, in apparent contradiction, both theoretical and experimental studies have shown that the π donor character of gold is usually far from negligible^34^–^36^ (especially toward carbon monoxide^37^) with important effects in catalysis.^38^,^39^ Recently, furthermore, a gold(i) complex showing Δ*ν*_CO_ < 0 has been fully characterized,^40^ bearing a neutral *o*-carborane diphosphine (DPCb) as an ancillary ligand. Such an “exception”, which is even more singular when considering that the formal positive charge should strengthen the CO bond, made the authors speak of “enhanced π back-donation” from the [(DPCb)Au]^+^ fragment. For the reader's convenience, an overview of the experimentally characterized systems, with the reported Δ*ν*_CO_ values and reference to the original papers, is displayed in Fig. 1. An additional gold(i) system, [{MeB[3-(Mes)Pz]_3_}Au(CO)], has been preliminarily reported as red-shifted in ref. 41.

The relationship of the DCD constituents of coordination bonds, determined unambiguously *via* charge-displacement (CD) analysis,^35^,^42^ with spectroscopic observables has been the subject of some of our recent work,^36^,^37^,^43^ and in the present work we have used this analysis to systematically study an extensive series of carbonyl compounds. The unique power of CD analysis lies in the fact that it provides a complete picture, across the entire molecular space, of the charge flow of σ and π character accompanying the formation of a coordination bond, and it permits a well-defined, consistent measure of the charge transfer (CT) associated both with the DCD components of the M–CO bond and with the σ and π components of the polarization^44^ taking place at the CO ligand itself. As a result, as we hope we will have convinced the reader by the end of the paper, this work provides a definitive and quantitative account of the role and interplay of the DCD components of the M–CO bond and of CO polarization in driving CO stretching response to coordination.

We thus investigate the relation between Δ*ν*_CO_ and Δ*r*_CO_ and the charge displacements of σ and π symmetry along the M–C–O axis in response to the M–CO bond formation in metal carbonyl complexes. We carry out our analysis first on an exhaustive series of 23 gold(i) carbonyls of formula [(L)Au(CO)]^0/+^, where L is a varying auxiliary ligand (including none), which includes 8 of the experimentally characterized complexes and which is evenly partitioned between charged and neutral complexes, as well as between classical (CO bond elongated and frequency red-shifted) and non-classical (CO bond shortened and frequency blue-shifted). The choice of binary gold complexes seems to be particularly simple and useful, as it permits to isolate and study systematically the effect of the *trans* ligand across a wide variety of metal binding properties and electronic effects. We begin our analysis (Section 3.1) by studying in greater detail the two extreme cases of “naked” Au^+^, [Au(CO)]^+^, which displays the experimentally largest blue-shift, and of [(DPCb)Au(CO)]^+^, which is the only known case of a positively charged but significantly red-shifted gold(i) complex. Having thus highlighted the main findings, we then thoroughly confirm them by extending the study to the whole series of complexes (Section 3.2). To complete the work we then also investigate the role of the metal itself in driving CO response to coordination, by studying a series of homoleptic [(CO)_*n*_M(CO)]^*m*^ complexes, with M including Hg, Ir, Ni, Fe, Cr, Mo, Co, Ru (Section 3.3). Finally, an *ad hoc* study of CO in a uniform axial electric field (Section 3.4) concludes the work, in order to isolate the impact of CO polarization and of its σ and π components on CO stretching response.

## 2 Methodology and computational details

In the charge-displacement (CD) analysis framework, a chemical bond A–B is analyzed in terms of the difference Δ*ρ*(*x*,*y*,*z*) between the electron density of the adduct AB and that of the two non-interacting fragments A and B frozen at their in-adduct geometries. A partial progressive integration of Δ*ρ*(*x*,*y*,*z*) along a suitably chosen bond axis *z* yields the so called charge-displacement function (CDF)^42^
1

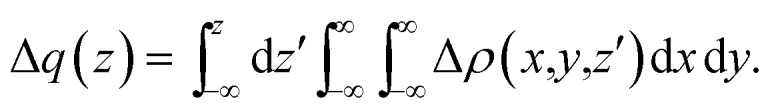




The CDF defines, at each point *z*, the exact amount of electron charge displaced from right to left (the direction of decreasing *z*) upon bond formation through a plane perpendicular to the *z* axis through the point *z* (negative CDF values indicate a charge flow in the opposite direction). If both the adduct and its constituting fragments have proper symmetry, Δ*ρ*(*x*,*y*,*z*) can be decomposed into additive components of σ and π symmetry with respect to the bond axis *z* (see ref. 35 for further details).

All of the complexes studied in this work have general formula [(L)_*n*_M(CO)]^*m*^. Since the M–CO bond is under investigation, the appropriate fragments are the ligand–metal moiety [(L)_*n*_M]^*m*^ and carbon monoxide CO, and the *z* reference axis joins the M and C centres. For the purpose of separating the σ and π components of Δ*ρ*(*x*,*y*,*z*), we group the orbitals of adduct and fragments according to the irreducible representations of the complex (and fragments) symmetry groups, which in the present cases are either the *C*_2v_ group (where the A_1_ representation corresponds to σ, while B_1_ and B_2_ correspond to π) or the *C*_3v_ group (A_1_ corresponding to σ donation, and E_1_ and E_2_ to π back-donation). No CO orbital is of A_2_ symmetry, therefore this representation is not relevant for the DCD analysis of the M–CO bond and is found to represent only a (minor) rearrangement internal to the ligand–metal fragment. Among the gold(i) complexes considered, [(PF_3_)Au(CO)]^+^, [(PH_3_)Au(CO)]^+^, [(P(CH_3_)_3_)Au(CO)]^+^, [(CF_3_)Au(CO)] and [(CH_3_)Au(CO)] belong to the *C*_3v_ point group. All others have *C*_2v_ symmetry. The symmetry point groups of the homoleptic complexes considered in Section 3.3 are listed in Table 2. The reduced symmetries *C*_2v_ and *C*_3v_ have been used to separate the σ and π components of the electron density difference also for these complexes.

The CDFs of the σ and π components of Δ*ρ*(*x*,*y*,*z*) provide a thorough, spatially detailed picture of the DCD donation and back-donation charge flows.^35^ Well-defined measures of the net charge transfer and of its donation and back-donation contributions (hereafter CT_net_, CTσdon and CTπback, respectively) can be obtained by taking the CDFs values at a plausible inter-fragment boundary, which we take to be the *z* point where equal-valued isodensity surfaces of the fragments become tangent.^35^,^36^

As mentioned in the Introduction, in the present context the CDF also provides precious additional information concerning CO polarization. Since the C–O bond is collinear with the M–C *z* axis of integration, the CDF in the C–O bond region represents the electron displacement within CO with respect to free CO in response to the M–CO bond formation. The amount of charge flowing across a plane normal to the CO bond through its mid-point (*i.e.*, the CDF value at *z* = *r*_CO_/2) can be usefully taken as a quantitative estimate of such polarization, and the total value can again itself be decomposed in σ and π components. We shall refer to these values as to CT_*r*_CO_/2_, 
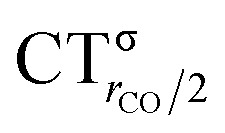
 and 
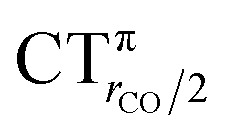
, respectively.

Geometry optimizations and the calculation of harmonic frequencies and electron densities were carried out by means of Density Functional Theory (DFT) with the ADF package.^45^–^47^ Becke's exchange functional^48^ in combination with the Lee–Yang–Parr correlation functional^49^ (BLYP) was adopted. We used an all electron triple-zeta basis set with two polarization functions (TZ2P) and a small frozen core for all atoms. Relativistic effects were included *via* the zeroth-order regular approximation (ZORA) Hamiltonian.^50^–^52^ An assessment of the effect of the exchange–correlation functional and of the basis set on the CDF is given in the ESI,† where a comparison is also made with results from fully relativistic calculations carried out with a recently implemented parallel version of the Dirac–Kohn–Sham program BERTHA.^53^–^55^

The purely electrostatic effect on the CO charge rearrangement was investigated using a uniform axial electric field (see also ref. 56–58) orientated along the C–O bond axis *z* (more details are given in Section 3.4). The density difference Δ*ρ*(*x*,*y*,*z*) in this case was formulated as the electron density of CO in the presence of the electric field at the actual minimum energy configuration minus that of free CO at the same geometry.

## 3 Results and discussion

As mentioned in the Introduction, we first describe here a detailed investigation of the M–CO bond in [Au(CO)]^+^ and [(DPCb)Au(CO)]^+^ (Section 3.1). We then extend the analysis to a whole series of 21 [(L)Au(CO)]^0/+^ complexes (Section 3.2) and, finally, to a series of nine homoleptic complexes of general formula [(CO)_*n*_M(CO)]^*m*^ (Section 3.3). The full list of complexes considered is in Tables 1 and 2. The purely electrostatic effect is investigated in the last Section (3.4) where an analysis of CO in a uniform axial electric field is carried out.

Three of the experimentally characterized gold complexes, with ligands DPCb, [HB(3,5-(CF_3_)_2_Pz)_3_]^–^ and Mes_3_P do not satisfy the symmetry requirements discussed in Section 2. [(DPCb)Au(CO)]^+^, however, is only slightly asymmetric in its minimum configuration and has been here constrained to *C*_2v_ symmetry (the difference in energy with respect to the unconstrained optimized configuration is as small as 1 kcal mol^–1^). The other two have been excluded from our analysis because they are much more asymmetric and to constrain them to *C*_3v_ symmetry would probably alter their properties significantly.

As Table 1 shows, the experimental CO stretching frequency for the three complexes [(CF_3_)Au(CO)], [(Cl)Au(CO)] and [(Br)Au(CO)] (the first of which is measured in the solid state and the others in solution) is actually blue-shifted rather than red-shifted as the calculations consistently suggest for all the neutral systems (the computed *v*_free-CO_ is 2143 cm^–1^). Regarding this apparent inconsistency, Frenking *et al.* recently found that the experimental blue shift is actually due to the presence of intermolecular interactions and not to the properties of the single molecule.^59^ They proved this by computing the CO frequency of small aggregates of [(CF_3_)Au(CO)] and of [(Cl)Au(CO)] and finding that the frequency increases from smaller to larger values than that of free CO. Indeed, Au–Au interactions have been experimentally observed for these two complexes in the solid state^24^,^60^ and are likely to occur also in solution, especially for ligands with little steric hindrance. For this reason, and since experimental data are available only for a small subset of the complexes considered here, we shall base our discussion on the DFT values of Δ*ν*_CO_ and Δ*r*_CO_ (computed *r*_free-CO_ = 1.137 Å). In fact, we shall most often refer to the latter parameter only, because the non-uniform influence of vibrational mode coupling, and the more complicated CO vibration modes in the homoleptic carbonyls, make Δ*ν*_CO_ a less reliable parameter than Δ*r*_CO_ for a quantitative analysis of its relation with the M–CO bond characteristics.

**Table 1:** Computed Δ*ν*_CO_ (cm^–1^) and Δ*r*_CO_ (Å) and charge-transfer results (*e*) obtained from the CD analysis for the considered series of [(L)Au(CO)]^0/+^ complexes. In boldface, data for the experimentally observed complexes. Reference values are *ν*_free-CO_ = 2106 cm^–1^ (experimental: 2143 cm^–1^), *r*_free-CO_ = 1.137 Å. ^*a*^ The vibrational coupling between the CO and the ligand has been eliminated through isotopic substitution ([(C^28^N)Au(CO)] and [(^3^H)Au(CO)])

	Δ*ν*_CO_ (exp. Δ*ν*_CO_)	Δ*r*_CO_	CT_net_	CTσdon	CTπback	CT_*r*_CO_/2_	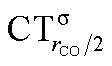	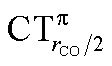
**Non classical behavior**								
**[(CO)Au(CO)]** ^**+**^	**72 (74 (** **ref. 28** **))**	**–0.010**	**0.08**	**0.21**	**–0.13**	**0.15**	**0.06**	**0.09**
[(PF_3_)Au(CO)]^+^	80	–0.009	0.09	0.22	–0.13	0.15	0.06	0.09
**[Au(CO)]** ^**+**^	**75 (94 (** **ref. 26** **))**	**–0.008**	**0.16**	**0.34**	**–0.18**	**0.16**	**0.07**	**0.09**
[(Ne)Au(CO)]^+^	76	–0.008	0.15	0.33	–0.18	0.16	0.07	0.09
[(C_2_H_4_)Au(CO)]^+^	63	–0.007	0.08	0.24	–0.16	0.13	0.06	0.07
[(PH_3_)Au(CO)]^+^	60	–0.007	0.06	0.22	–0.16	0.12	0.05	0.07
[(C_2_H_2_)Au(CO)]^+^	63	–0.007	0.06	0.23	–0.17	0.13	0.06	0.07
[(Xe)Au(CO)]^+^	62	–0.006	0.08	0.27	–0.19	0.13	0.06	0.07
[(P(CH_3_)_3_)Au(CO)]^+^	38	–0.004	0.05	0.22	–0.17	0.10	0.05	0.05
[(NHC)Au(CO)]^+^	39	–0.004	0.00	0.20	–0.20	0.10	0.05	0.05
[(C_5_H_5_N)Au(CO)]^+^	46	–0.003	0.01	0.23	–0.22	0.10	0.05	0.05
**[(SIdipp)Au(CO)]** ^**+**^	**23 (54 (** **ref. 30** **))**	**–0.002**	**–0.01**	**0.20**	**–0.21**	**0.08**	**0.05**	**0.03**
**[(Idipp)Au(CO)]** ^**+**^	**17 (49 (** **ref. 30** **))**	**–0.001**	**–0.02**	**0.20**	**–0.22**	**0.08**	**0.05**	**0.02**

**Classical behavior**								
**[(CF**_**3**_ **)Au(CO)]**	**–2 (51 (** **ref. 24** **))**	**0.002**	**–0.02**	**0.22**	**–0.24**	**0.05**	**0.05**	**0.00**
[(CN)Au(CO)]	–6^*a*^	0.003	–0.06	0.20	–0.26	0.05	0.05	0.00
[(H)Au(CO)]	–23^*a*^	0.004	–0.07	0.19	–0.26	0.02	0.04	–0.02
[(CH_3_)Au(CO)]	–42	0.007	–0.07	0.21	–0.28	0.01	0.04	–0.03
[(C_6_H_5_)Au(CO)]	–45	0.007	–0.06	0.22	–0.28	0.02	0.04	–0.02
[(I)Au(CO)]	–45	0.008	–0.08	0.24	–0.32	0.02	0.05	–0.03
**[(Cl)Au(CO)]**	**–29 (13 (** **ref. 27** **))**	**0.008**	**–0.11**	**0.23**	**–0.33**	**0.02**	**0.05**	**–0.03**
**[(Br)Au(CO)]**	**–40 (10 (** **ref. 27** **))**	**–0.008**	**–0.09**	**0.24**	**–0.33**	**0.02**	**0.05**	**–0.03**
[(F)Au(CO)]	–23	0.009	–0.13	0.22	–0.35	0.02	0.05	–0.04
**[(DPCb)Au(CO)]** ^**+**^	**–76 (–30 (** **ref. 40** **))**	**0.010**	**–0.06**	**0.26**	**–0.32**	**0.03**	**0.05**	**–0.02**

### 3.1 [Au(CO)]^+^ and [(DPCb)Au(CO)]^+^

We start our analysis with an in-depth investigation of the gold carbon coordination bond in [Au(CO)]^+^ and [(DPCb)Au(CO)]^+^. As mentioned in the Introduction, among the experimentally characterized gold carbonyl complexes, these two systems display the most different spectroscopic properties. [Au(CO)]^+^ (observed in neon matrix^26^) shows a CO stretching frequency much higher than that of free CO (experimental Δ*ν*_CO_ = 94 cm^–1^) while [(DPCb)Au(CO)]^+^ (ref. 40) represents a unique case of cationic complex with red-shifted CO stretching frequency (experimental Δ*ν*_CO_ = –30 cm^–1^). The computed values (Δ*ν*_CO_ = 75 cm^–1^ and –76 cm^–1^, respectively, see Table 1) reflect this opposite behavior.

**Fig. 2:**
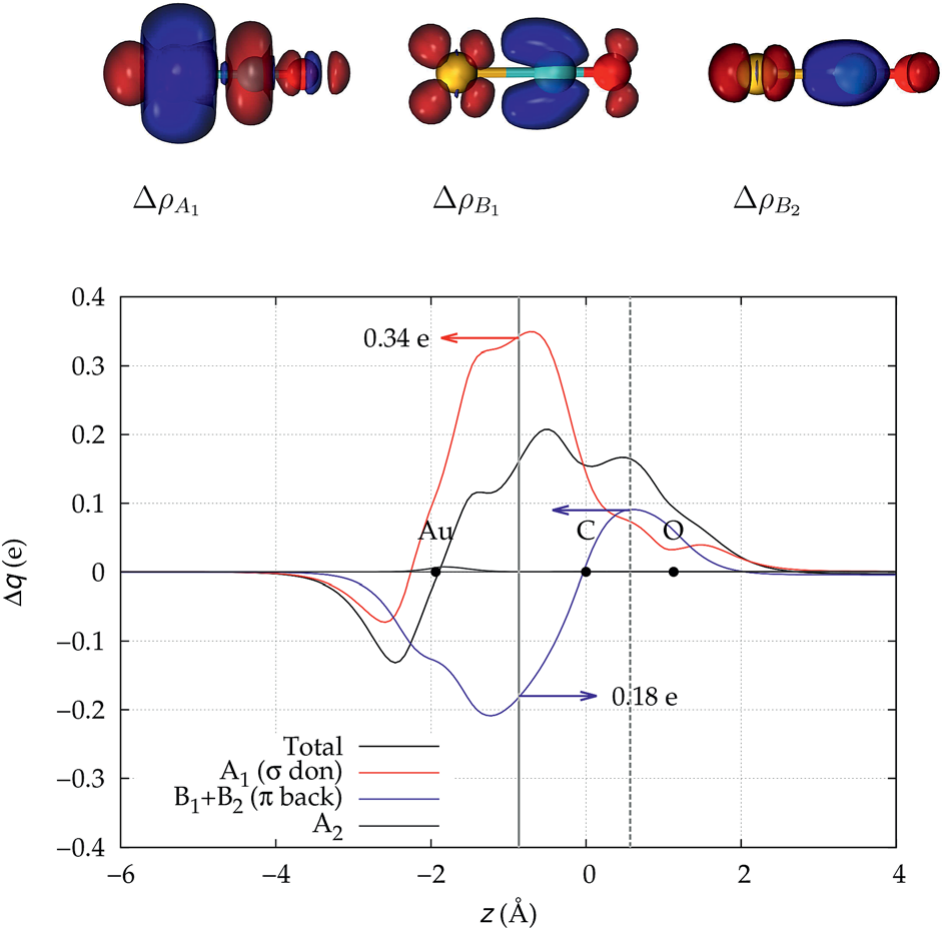
Total CDF and its symmetry (*C*_2v_) components for the Au–CO bond in the complex [Au(CO)]^+^. Black dots indicate the *z* position of the atomic nuclei. A solid vertical line marks the boundary between the Au^+^ and the CO fragments (see Section 2 for its definition). A dashed vertical line indicates the midpoint of the C–O bond (*z* = *r*_CO_/2). At the top: isodensity surfaces (±0.0025 e a.u.^–1^ (ref. 3)) for the A_1_, B_1_ and B_2_ components of Δ*ρ*(*x*,*y*,*z*). Red surfaces identify charge depletion areas, blue surfaces identify charge accumulation areas.

We focus first on [Au(CO)]^+^, showing in Fig. 2 the CDFs for the overall density difference and its symmetry-separated components. We recall here that, at a given point *z*, a positive CDF value corresponds to a charge flow from right to left (*i.e.*, in the Au^+^ ← CO direction) while a negative value corresponds to a charge flow in the opposite (Au^+^ → CO) direction. The total CDF is positive over both the Au–C and C–O bond regions and also at the oxygen far side of CO, indicating a continuous flow of electrons in the direction from CO towards gold. The negative values of the curve on the left side of Au^+^ indicate a rearrangement in the opposite direction, which was shown in ref. 42 to be due to gold sd hybridization. The total CDF results from an A_1_ component which is large and positive in the Au–carbon region (identifying σ donation) and a B_1_ + B_2_ component which is negative in the same zone (identifying π back-donation) plus a negligible A_2_ component. These components are easily recognized in the isodensity plots of the respective density difference shown at the top of the figure.

The net charge transfer CT_net_ from CO to Au^+^ (the CDF value at the boundary solid vertical line) amounts to 0.16*e* resulting from a donation component CTσdon of 0.34*e* and a back-donation component CTπback of 0.18*e*. The first important comment here is that, in a system like this showing a large blue-shift of the CO stretching frequency, back-donation is actually a significant component of the interaction, estimated to be more than half as large as the donation.

An analogous significant contribution from the electron charge rearrangement of π symmetry was also recently highlighted in ref. 29 through a Natural Orbitals for Chemical Valence-Extended Transition State (NOCV-ETS)^61^ energy decomposition analysis. In particular, the π contribution to the overall orbital interaction energy Δ*E*_orb_ was found to be surprisingly large (32.5% of the overall Δ*E*_orb_). The authors were cautious, however, in attributing such contribution exclusively to π back-donation, as Δ*E*_orb_ not only accounts for genuine inter-fragment orbital interactions but also for the polarization of the orbitals within each fragment.

This uncertainty may be dissolved here, because, as discussed in Section 2, the interfragment charge transfer and its components are automatically separated from the corresponding components of CO polarization in the CDF picture. Inspection of Fig. 2 is in fact particularly revealing in this respect. Focusing on the CDFs in the carbonyl region, we notice immediately that the positive value of the total function indicates that the CO bond is on the whole polarized in the C ← O direction. Remarkably, this polarization results from the concordant positive contributions of both the σ and π components. We indeed see that, while the σ CDF keeps its (positive) sign on the right hand side of C and even beyond the oxygen site, an inversion (from negative to positive) is seen to occur for the π component precisely at the carbon site, leading to a maximum located at about the mid-point of the C–O bond. In both cases, therefore, there is a displacement of electrons from oxygen towards carbon, which is due to the presence of the positively charged metal fragment. As discussed in Section 2, we can quantify the extent of CO polarization by taking the CDFs values at the mid-point of the CO bond (dashed vertical line in Fig. 2). For the case under examination, the C ← O polarization amounts to CT_*r*_CO_/2_ = 0.16*e*, resulting from a σ contribution 
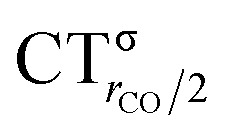
 of 0.07*e* and a π contribution 
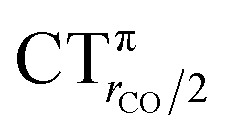
 of 0.09.

**Fig. 3:**
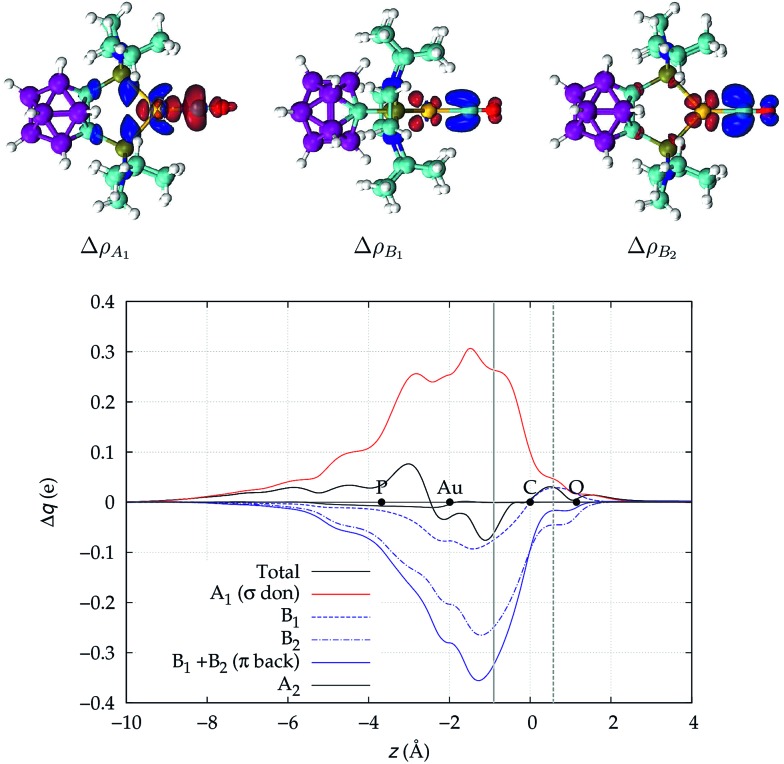
Total CDF and its symmetry (*C*_2v_) components for the Au–CO bond in the complex [(DPCb)Au(CO)]^+^. Black dots indicate the *z* position of the atomic nuclei. A solid vertical line marks the boundary between the [(DPCb)Au]^+^ and the CO fragments (see Section 2 for its definition). A dashed vertical line indicates the midpoint of the C–O bond (*z* = *r*__CO__/2). At the top: isodensity surfaces (±0.0025 e a.u.^–1^ (ref. 3)) for the A_1_, B_1_ and B_2_ components of Δ*ρ*(*x*,*y*,*z*). Red surfaces identify charge depletion areas, blue surfaces identify charge accumulation areas.

We now turn to [(DPCb)Au(CO)]^+^, with its CDFs reported in Fig. 3. This is analogous to Fig. 2 except that here the B_1_ (dashed blue line) and B_2_ (dotted-dashed line) components are not identical and are shown separately in the plot. We notice an immediate striking contrast with the previous [Au(CO)]^+^ case, in that the back-donation components globally dominate over σ donation in the coordination bond region, so that the total CDF is negative everywhere, indicating a continuous, though modest, flow of electrons from [(DPCb)Au]^+^ to CO. This confirms the already cited findings of ref. 40. We note that π back-donation is in turn largely dominated by the B_2_ component. The net charge transfer at the inter-fragment boundary is –0.06*e*, resulting from a σ donation component of 0.26*e* (A_1_) and a π back-donation component of –0.32*e* (–0.07 due to the B_1_ component and –0.25 due to the B_2_ component).

The polarization of the electron cloud in the carbonyl region also differs remarkably from that in [Au(CO)]^+^. In analogy with [Au(CO)]^+^, the σ CDF remains positive in the CO region and the B_1_ component turns positive at the C site, reflecting the polarization of the CO bonding orbitals due to the electrostatic effect of the metal fragment. However, by contrast, the B_2_ component maintains its negative sign also in the CO region, *i.e.* the back-donation it represents is so pronounced that it penetrates the CO region and extends even beyond the oxygen. As a consequence, the CO bond is on the whole slightly polarized in the C ← O direction (CT_*r*_CO_/2_ = 0.03*e*), resulting from a σ polarization in the same direction 
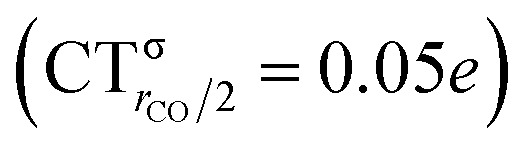
 and a π polarization in the opposite C → O direction 
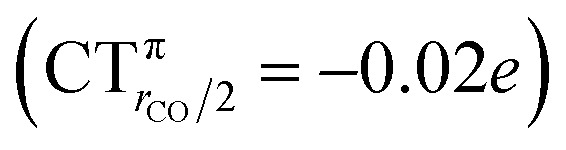
.

It is worth deepening the comparison between the two complexes examined so far. In both, the metallic fragment bears a formal positive charge. However, [Au(CO)]^+^ behaves non-classically (blue-shifted Δ*ν*_CO_), while [(DPCb)Au(CO)]^+^ behaves classically (red-shifted Δ*ν*_CO_). The CD analysis reveals that the σ donation component of the metal–CO bond is roughly comparable in the two cases (CTσdon 0.34 *vs.* 0.26*e*), while π back-donation is almost twice as large in [(DPCb)Au(CO)]^+^ (CTπback 0.32 *vs.* 0.18*e*) and its extent substantially reduces the C ← O polarization of the CO bond. The polarization of the CO σ bonding orbitals is comparable in the two complexes (
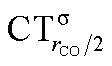
 0.07 *vs.* 0.05*e*), but that of the π bonding orbitals is not (
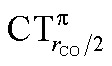
 0.09 *vs.* –0.02*e*). These findings suggest that π electron displacement upon coordination is the main factor driving CO bond response. In particular, if the presence of the metal fragment, especially if positively charged, is capable of polarizing the π CO bonding orbitals, even in the presence of a significant back-donation, the CO bond is strengthened; if, on the other hand, π back-donation is strong and extended enough to contrast CO polarization, even in the presence of an equally cationic metal fragment, the CO bond is weakened.

### 3.2 The complete [(L)Au(CO)]^0/+^ series

**Fig. 4:**
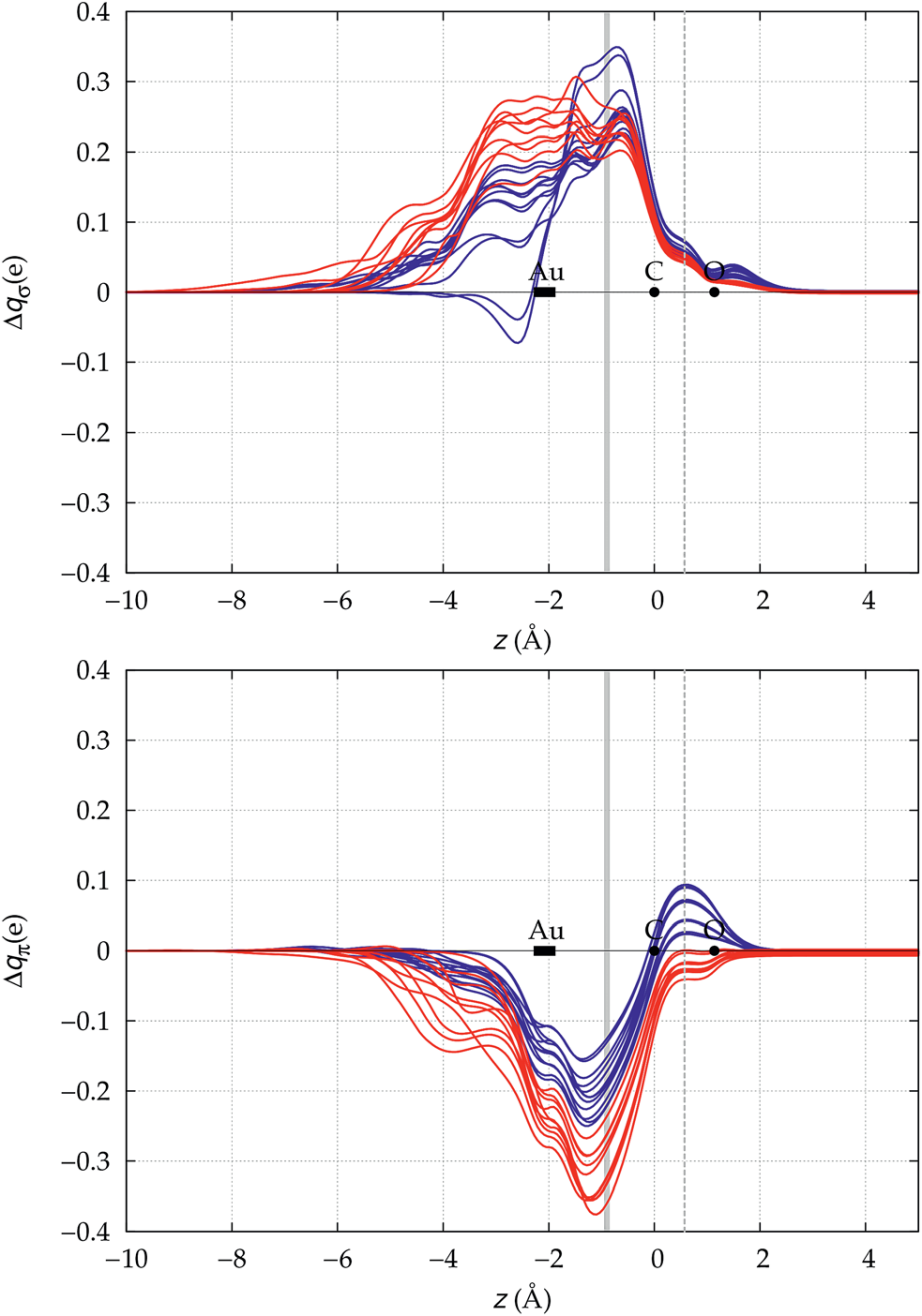
σ donation (top panel) and π back-donation (bottom panel) CDFs for the Au–CO bond in the series of [(L)Au(CO)]^0/+^ complexes of Table 1. Red lines (blue lines) are used for complexes showing red-shift (blue-shift) in the computed Δ*ν*_CO_. The *z* origin is placed at the C atom for all complexes and black dots indicate the position of C and O (the latter varying negligibly, less than 0.02 Å, among the complexes). A dashed vertical line marks the C–O midpoint. The position of the Au atom across the series varies more significantly and its range is marked by a rectangle. Similarly, a gray vertical band indicates the range of the interfragment boundary.

We now need to verify if the above preliminary surmise stands the test of a wider series of carbonyl compounds. To this end, we have extended the analysis to all 23 [(L)Au(CO)]^0/+^ complexes listed in Table 1, which collects the spectroscopic data for Δ*ν*_CO_ and Δ*r*_CO_ as well as the various computed CT figures. The complexes are listed in order of increasing Δ*r*_CO_ and the experimentally characterized compounds are those shown in boldface. As briefly discussed at the beginning of Section 3, it is seen that, according to our computed shifts, the neutral complexes plus [(DPCb)Au(CO)]^+^ behave classically, while the remaining cationic complexes behave non-classically. The σ donation and π back-donation CDFs for these complexes are collected, respectively, in the top and bottom panel of Fig. 4. Red lines are for the complexes showing red shift of *ν*_CO_, blue lines are for those showing blue shift.

Two eye-catching features emerge upon inspection of Fig. 4. The first is that all systems exhibit a surprisingly similar σ charge rearrangement (top panel) in the CO fragment region, in contrast with a much wider variability on the metal fragment side and despite the fact that some of the complexes are neutral and some cationic. In fact, as Table 1 shows, if one excludes the special cases of the naked Au^+^, of the inert ligands Ne and Xe, and of the anomalous [(DPCb)Au(CO)]^+^, even the net ligand-to-metal σ donation, CTσdon, varies by only 0.05*e* across the whole series of ligands. On the contrary, the π CDF (bottom panel of Fig. 4) appears to be strongly influenced by the nature of the ligand over the whole molecular region, and CTπback varies by 0.22*e* over the ligand series. The second important observation is that, in the CO region, the complexes showing a blue-shifted *ν*_CO_ (blue lines) all invariably exhibit a flow of π electrons in the C ← O direction 
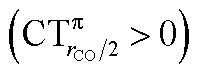
, due to the positively charged metallic fragment, while the complexes showing red-shifted *ν*_CO_ (red lines) exhibit a negative 
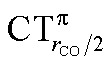
, *i.e.*, charge flows in the opposite C → O direction (with the exception of two complexes for which 
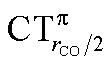
 is essentially vanishing and the red-shift is also negligibly small).

**Fig. 5:**
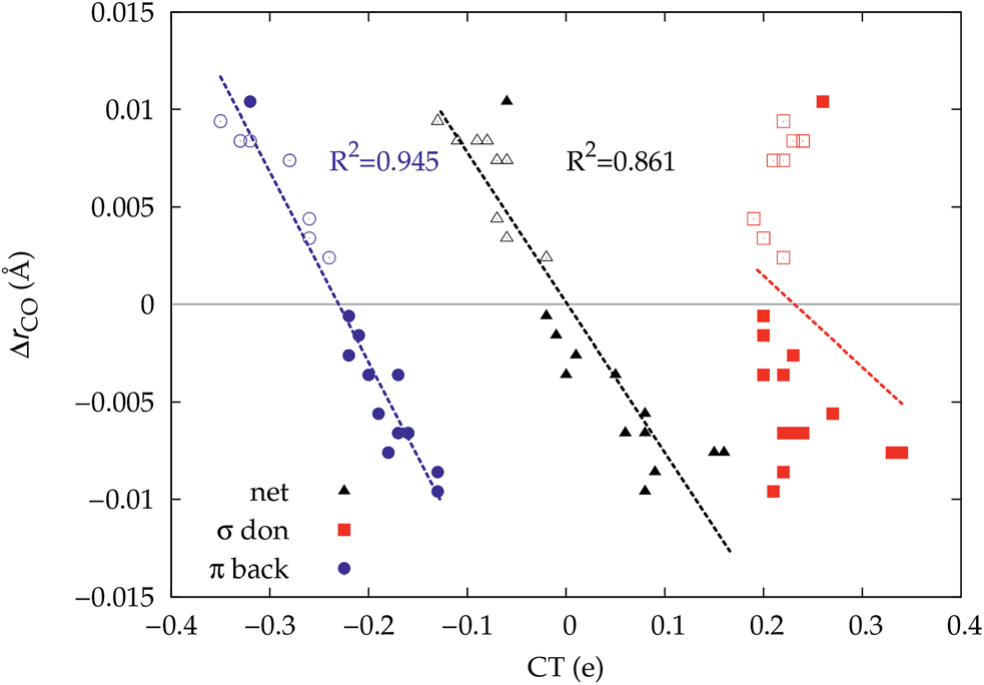
Correlation between the computed Δ*r*_CO_ in the considered series of [(L)Au(CO)]^0/+^ complexes and the CT_net_ (black triangles), CTσdon (red squares) and CTπback (blue circles). Empty symbols are for the neutral species, filled symbols for the cationic species.

**Fig. 6:**
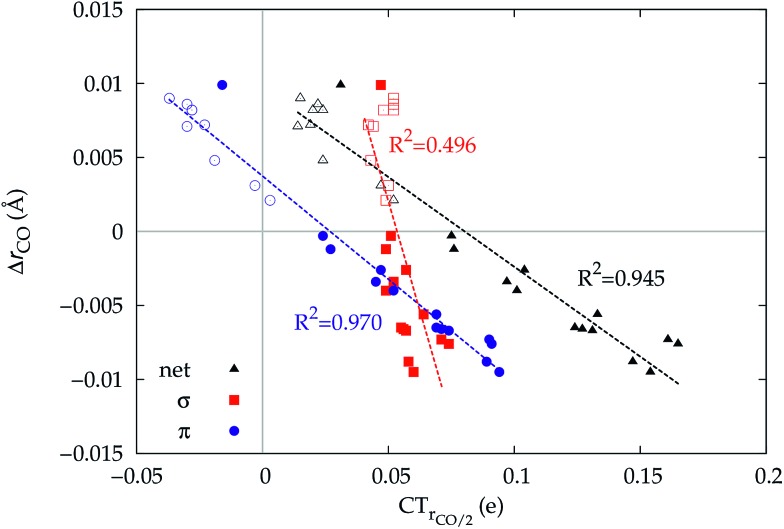
Correlation between the computed Δ*r*_CO_ in the considered series of [(L)Au(CO)]^0/+^ complexes and CT_*r*_CO_/2_ (black triangles), 
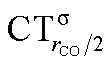
 (red squares) and 
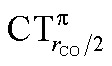
 (blue circles). Empty symbols are for the neutral species, filled symbols for the cationic species.

It thus appears quite clearly that in the series of gold(i) carbonyls: (i) σ donation is much less tunable than π back-donation, being very little dependent on the nature and the charge of the ligand; (ii) whereas the net CO bond polarization turns out to be invariably oriented in the C ← O direction (CT_*r*_CO_/2_ > 0), the direction of its π density component can vary and appears to be tightly connected with the direction of the CO stretching shift and bond-length change. These findings are given a definitive illustration in Fig. 5 and 6 where the correlation of Δ*r*_CO_ with CT_net_, CTσdon, CTπback, CT_*r*_CO_/2_, 
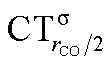
 and 
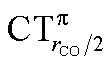
 is reported. In both figures, black triangles are used for the overall CT, red squares for its σ component and blue circles for its π component. Empty symbols are for the neutral species, filled ones are for the cationic species.

Focusing first on Fig. 5, no correlation is found, as expected, between Δ*r*_CO_ and CTσdon, while a good inverse correlation (*R*^2^ = 0.945) can be seen between Δ*r*_CO_ and CTπback, a trace of which remains in the plot of Δ*r*_CO_*vs.* CT_net_. The same bond weakening effect of π back-donation is also evident in the plot of Δ*ν*_CO_*vs.* CTπback (see ESI†), though correlation, as mentioned above, is made worse by mode coupling (*R*^2^ = 0.849). Fig. 6 shows the correlation of Δ*r*_CO_ with CT_*r*_CO_/2_ and its components 
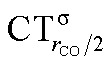
 and 
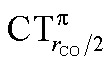
. Not surprisingly, as these quantities are all directly related to the charge rearrangement of the CO bond itself, correlations are here quantitatively better (*R*^2^ is 0.970 for that with 
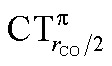
). Obviously, as Δ*r*_CO_ correlates well with both π back-donation and CO π electron polarization, the latter two quantities are also in mutual correlation.

### 3.3 Homoleptic complexes: the [(CO)_*n*_M(CO)]^*m*^ series

In the previous sections we considered a series of gold(i) complexes where the donor/acceptor properties of the M–CO bond were varied through the ligand L. We now extend the analysis to a series of homoleptic carbonyls of formula [(CO)_*n*_M(CO)]^*m*^, where the relative extent of the DCD constituents of the M–CO bond and CO polarization are varied essentially by changing the metal. The full list of the considered homoleptic complexes is in Table 2, reporting their spectroscopic shifts and CD decomposition results. Complexes are listed in order of increasing value of Δ*r*_CO_. We omit for brevity a presentation of the complete CDFs. The computed structures for these systems are in agreement with experimental X-ray data where available.^62^–^66^ Hg(CO)_2_^2+^ and Ir(CO)_6_^3+^, both cationic, behave non classically, with experimental blue-shifted *ν*_CO_ at 2279.5 cm^–1^ for the former and at 2254, 2276 and 2298 cm^–1^ for the latter.^65^,^66^ On the opposite side, the anionic complexes show exceptionally low CO stretching frequency, the most red-shifted being that of Fe(CO)_4_^2–^ at 1730 cm^–1^ (this is the first anionic carbonyl complex spectroscopically observed^67^,^68^). In between are Mo(CO)_6_, Fe(CO)_5_ (for which both the axial and equatorial M–CO bonds have been investigated),^69^ Ni(CO)_4_ and Cr(CO)_6_. The complexes present therefore a wide range of *ν*_CO_ variation but Δ*ν*_CO_ turns out not to be a good parameter for analyzing correlations with the CD data because normal-mode coupling varies significantly with the different structure of the complexes. We therefore base our discussion, as already done for the gold(i) complexes, on the computed Δ*r*_CO_. This varies in a range of 0.087 Å over the series, from –0.018 to 0.069 Å (Table 2).

**Table 2:** Symmetry group, experimental Δ*ν*_CO_ (and related symmetry mode), computed Δ*r*_CO_ and results obtained from the CD analysis for the considered series of homoleptic carbonyl complexes [(CO)_*n*_M(CO)]^*m*^. Distances in Å, charge transfers in *e*

	Sym.	Exp. Δ*ν*_CO_	Δ*r*_CO_	CT_net_	CTσdon	CTπback	CT_*r*_CO_/2_	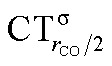	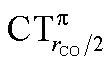
**Non classical behavior**									
Hg(CO)_2_^2+^	C_∞v_	136 (ref. 66) (A_1_)	–0.018	0.29	0.31	–0.02	0.13	0.08	0.21
Ir(CO)_6_^3+^	*O*_h_	155 (ref. 65) (A_1g_)	–0.015	0.17	0.31	–0.13	0.08	0.10	0.18
		133 (ref. 65) (E_g_)							
		111 (ref. 65) (T_1u_)							

**Classical behavior**									
Ni(CO)_4_	*T*_d_	–23 (ref. 70) (A_1_)	0.012	–0.16	0.16	–0.32	–0.01	0.04	–0.05
Fe(CO)_5_(ax.)	*D*_3h_	–22 (ref. 69) (A′_1_)	0.014	–0.18	0.23	–0.41	–0.02	0.06	–0.08
		–101 (ref. 69) (A′_1_)							
		–109 (ref. 69) (A′′_2_)							
Cr(CO)_6_	*O*_h_	–24 (ref. 71) (A_1g_)	0.016	–0.21	0.17	–0.37	–0.02	0.05	–0.07
		–116 (ref. 71) (E_g_)							
		–143 (ref. 71) (T_1u_)							
Mo(CO)_6_	*O*_h_	–22 (ref. 71) (A_1g_)	0.016	–0.23	0.14	–0.37	–0.02	0.04	–0.06
		–118 (ref. 71) (E_g_)							
		–140 (ref. 71) (T_1u_)							
Fe(CO)_5_(eq.)	*D*_3h_	–22 (ref. 69) (A′_1_)	0.018	–0.20	0.20	–0.40	–0.03	0.05	–0.08
Co(CO)_4_^–^	*T*_d_	–255 (ref. 72) (A_1_)	0.038	–0.36	0.16	–0.52	–0.15	0.03	–0.18
Ir(CO)_4_^–^	*T*_d_	–248 (ref. 73) (A_1_)	0.039	–0.28	0.30	–0.58	–0.14	0.04	–0.18
Ru(CO)_4_^2–^	*T*_d_	–407 (ref. 74) (A_1_)	0.066	–0.42	0.25	–0.67	–0.24	0.03	–0.27
Fe(CO)_4_^2–^	*T*_d_	–413 (ref. 75) (A_1_)	0.069	–0.55	0.16	–0.71	–0.28	0.02	–0.30

The table shows that also in this series of compounds the range of variation in π back-donation (0.69*e*) is much larger than that of σ donation (0.15*e*). In particular, almost no back-donation is found for Hg(CO)_2_^2+^ while CTπback for [Fe(CO)_4_]^2–^ is as high as 0.71*e*. This picture is consistent with the simple VB view discussed in the Introduction, in that we go from a purely σ M–CO bond (structure a) for Hg(CO)_2_^2+^ to a situation in which all π* CO orbitals are engaged in back-bonding (structure c) for [Fe(CO)_4_]^2–^. Also the charge rearrangement (polarization) in the carbonyl region is seen to follow a similar trend, with a much narrower range of 
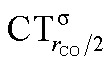
 values (between 0.02 and 0.10*e*) than that of 
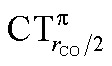
 (from 0.21 to –0.30*e*). As before, no clear correlation can be discerned between Δ*r*_CO_ and the σ CT data, while CTπback and 
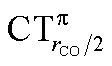
 values are seen to decrease almost monotonically as Δ*r*_CO_ increases.

**Fig. 7:**
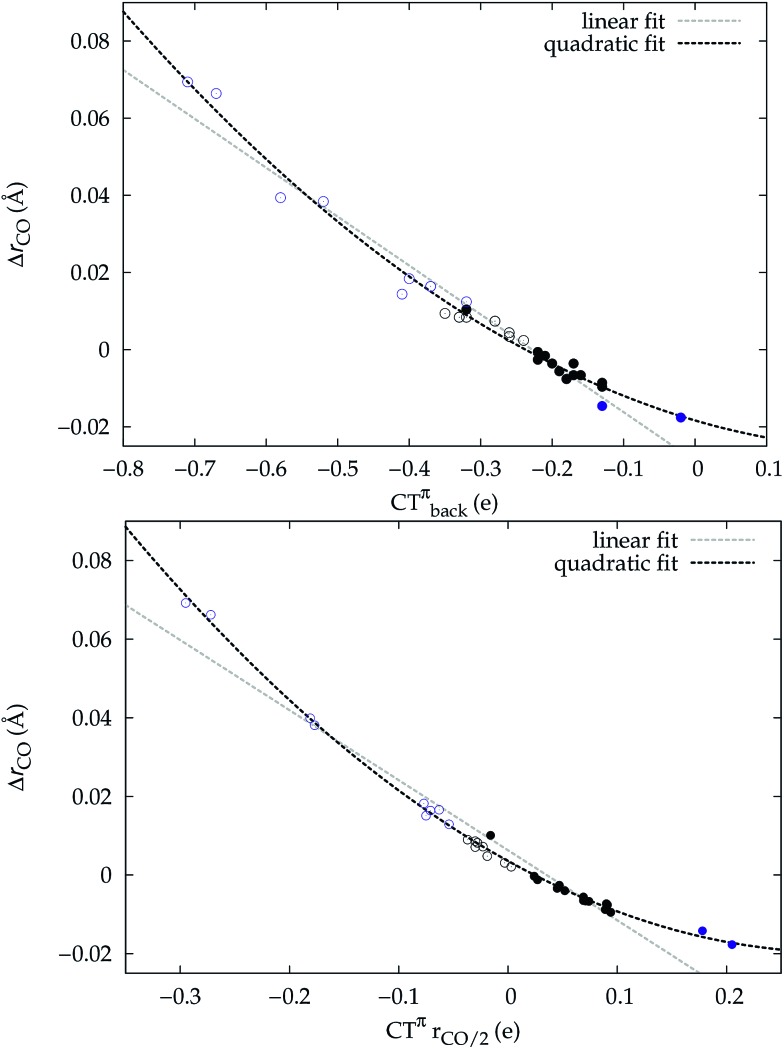
Correlation between CO bond-length change upon coordination, Δ*r*_CO_, and (a) M → CO π back-donation CTπback (upper panel) (b) CO π electron polarization 
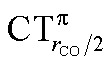
 (lower panel), for the whole set of complexes studied. Black-colored points refer to the gold(i) complexes, blue-colored points to the homoleptic carbonyls (filled circles are for positively charged complexes, empty circles for neutral or negatively charged ones). Both the linear fit (*R*^2^ = 0.959 for the upper panel, *R*^2^ = 0.945 for the lower panel) and the quadratic fit (*R*^2^ = 0.984 for the upper panel and *R*^2^ = 0.996 for the lower panel) curves are shown.

A plot of Δ*r*_CO_*vs.* either CTπback or 
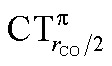
 for the whole set of complexes studied, including the present homoleptic carbonyls in addition to the gold(i) series, appears in fact to suggest, because the range of variation is now significantly enlarged, that a quadratic fit, rather than a linear one, may better represent the actual correlation (an evident non-linear relationship has already been found between the electric field strength and Δ*r*_CO_^56^). Fig. 7 very clearly shows this to be the case, with the accuracy of all fits improved with respect to the sole subset of gold complexes.

Once again, in the homoleptic series, the carbonyl complexes featuring CO bond strengthening (blue-shifted Δ*ν*_CO_ and negative Δ*r*_CO_), *i.e.* the cationic Hg(CO)_2_^2+^ and Ir(CO)_6_^3+^, show a flow of π electrons in the C ← O direction. All other complexes, where the CO bond weakens (red-shifted Δ*ν*_CO_ and positive Δ*r*_CO_) show opposite-direction flows.

### 3.4 CO in a uniform axial electric field

The observation that the CO bond is lengthened or shortened upon formation of the M–CO bond according to whether the CO bonding orbitals of π symmetry are polarized in the C → O or C ← O direction, respectively, is certainly remarkable. To verify that this is a general fact, actually independent of CO coordination, we discuss in this last section an *ad hoc* study of the electron cloud rearrangement and stretching response of CO in an external uniform axial electric field oriented along the C–O bond axis.

**Fig. 8:**
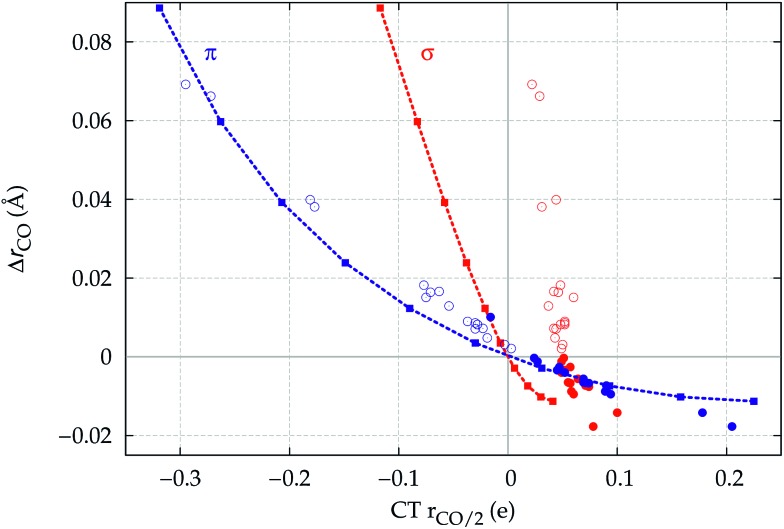
The dashed lines show Δ*r*_CO_*versus* the σ (red color) and π (blue) CO polarization CT_*r*_CO_/2_ for a CO molecule placed in a uniform axial electric field of magnitude ranging from –0.11 to 0.07 a.u. in steps of 0.02 a.u. (colored square points). For comparison, also shown is the correlation between Δ*r*_CO_ and σ and π CO polarization (red and blue circles, respectively) for the whole series of Au and homoleptic complexes studied. The empty circles are for neutral or negatively charged complexes, the filled circles for cationic ones.

In Fig. 8, we show the computed CO stretching Δ*r*_CO_ reported *versus* the π and σ components of CT_*r*_CO_/2_. The latter vary as a result of the applied field in the same figure. The points representing the computed Δ*r*_CO_ and π and σ components of CT_*r*_CO_/2_ are reported for the whole series of carbonyl complexes studied in this work.

Let us focus first on the stretching response to the electric field. When the field is absent, the system corresponds to free CO and Δ*r*_CO_, 
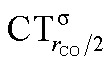
 and 
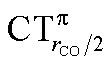
 are all zero. As the field increases on the left, in the direction that induces (linearly) C → O (negative) polarization, C–O bond length increases quadratically and π polarization is seen to increase much more rapidly than σ polarization. Conversely, as the field increases on the right, inducing C ← O polarization, the C–O bond shortens (much less rapidly).

When we now compare these curves with the relation observed between Δ*r*_CO_ and the σ and π components of CO polarization induced by metal coordination, rather than by an applied field (disconnected circles in the figure), we notice immediately that the π circles follow quite closely the correlation between field-induced polarization and stretching, while, in striking contrast, the σ circles deviate from the field-induced line (a clear indication of a much more pronounced “chemical” signature) and, moreover, span a very narrow range of (positive) polarization, essentially without any correlation with the widely varying Δ*r*_CO_. This is indeed a very strong confirmation that the CO stretching response to any solicitation causing electron charge rearrangement, be it the formation of a M–CO coordination bond or the effect of an external electric field, is driven essentially exclusively by the charge rearrangement of π symmetry: whether induced by an external electric field or by metal coordination, C → O (C ← O) polarization of the π bond orbitals invariably and tightly correlates with bond lengthening (shortening).

## 4 Conclusions

In this work we have carried out an in-depth analysis of the M–CO bond in [(L)_*n*_M(CO)]^*m*^ metal carbonyl complexes, with the aim of elucidating on quantitative grounds the σ donation and π back-donation effects on the CO stretching response, in particular the change in bond length Δ*r*_CO_, to the M–CO bond formation. The analysis was carried out for a large variety of carbonyls, in which the relative extent of the DCD constituents were varied both through L in a series of [(L)Au(CO)]^0/+^ gold(i) carbonyl complexes and through M in a series of anionic, neutral and cationic [(CO)_*n*_M(CO)]^*m*^ homoleptic carbonyls. Crucially, for the purpose of this investigation, reliable and consistent measures, not only of σ donation and π back-donation charges but also of the σ and π components of CO polarization were obtained by the well-established charge-displacement analysis of electron-density differences, as resulting from accurate DFT calculations. The nature of the M–CO bond in the considered complexes was found to range smoothly between the two extreme cases of an almost purely σ bonded complex (Hg(CO)_2_^2+^, CTπback = 0.02*e*) and of a strongly back-bonded complex ([Fe(CO)_4_]^2–^, CTπback = 0.71*e*). Conversely, all complexes were found to feature a narrowly comparable σ donation component, with CTσdon values ranging from 0.14 to 0.34*e*. The same picture holds accurately for the electron cloud rearrangement over the carbonyl region: all considered complexes feature a comparable σ polarization of CO and a much more variable π polarization. Quite remarkably, no correlation is found between Δ*r*_CO_ and the σ displacements, while Δ*r*_CO_, π back-donation and CO π polarization all correlate tightly with one another.

These results show that the driving force of the CO stretching response to the M–CO bond formation is provided exclusively by the changes taking place in the π electron density. In the complexes studied, such π charge rearrangement is found to result from the interplay between π back-donation (structures a–c of the Introduction) and the electrostatic effect (structures d–f) exerted by the metal–ligand fragment. In particular, cationic metal–ligand fragments polarize the π CO bonding orbitals in the C ← O direction, thus shortening the bond and enhancing the covalency, as highlighted in ref. 23. This effect, on the other hand, is contrasted by π back-donation shifting charge in the opposite direction. The net direction C ← O or C → O of the polarization of π CO bonding orbitals is found to invariably determine whether the CO bond is strengthened or weakened, respectively. This is most evident in the [(DPCb)Au(CO)]^+^ complex, where π back-donation is so strong as to invert the polarization of the π CO bonding orbitals in the C → O direction despite the formal positive charge on the ligand–metal fragment, making it the only example of a cationic gold(i) carbonyl complex with classical behavior (Δ*r*_CO_ > 0). An *ad hoc* study of CO in a uniform axial electric field demonstrates that it is indeed the polarization of the π CO bonding orbitals, no matter how induced (whether by the coordination bond to M or by an electric field), that drives direction and magnitude of the CO stretching response to the M–CO bond formation.

Regarding the fundamental question of what can be inferred on the nature of the M–CO bond from the analysis of Δ*r*_CO_ (and less reliably, due to mode coupling, Δ*ν*_CO_) in metal carbonyl complexes, we conclude that the value of Δ*r*_CO_ quantifies to an excellent extent the π back-donation component of the M–CO bond, since such component directly correlates with the π polarization. In particular, where 
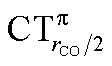
 changes its sign (*i.e.* the polarization of π CO bonding orbitals changes direction determining whether the CO bond is weakened or strengthened), CTπback is approximately as high as the average extent of σ donation among the complexes herein considered. This indicates that π back-donation is an important component also in the class of non-classical complexes, as those of gold(i) considered in this work.

## Supplementary Material

Supplementary information
