# Supplementary material for: How π back-donation quantitatively controls the CO stretching response in classical and non-classical metal carbonyl complexes
Source: Chem Sci. 2015 Oct 26;7(2):1174–84. doi: 10.1039/c5sc02971f (PMC5975789; doi:10.1039/c5sc02971f)
Supplement: Supplementary file 1 [file SC-007-C5SC02971F-s001.pdf]

Electronic Supplementary Material (ESI) for Chemical Science.
This journal is © The Royal Society of Chemistry 2015

Electronic Supplementary Information

How π back-donation quantitatively controls the CO stretching response in classical and non-classical metal carbonyl complexes

Giovanni Bistoni,*[ab] Sergio Rampino,*[b] Nicola Scafuri,[c] Gianluca Ciancaleoni,[b] Daniele Zuccaccia,[d] Leonardo Belpassi,[b] and Francesco Tarantelli*[ab]

[a] *Dipartimento di Chimica, Biologia e Biotecnologie, Università di Perugia, Via Elce di Sotto 8, 06123 Perugia, Italy*
[b] *Istituto di Scienze e Tecnologie Molecolari del CNR, Via Elce di Sotto 8, 06123 Perugia, Italy*
[c] *Institut Charles Gerhardt, Université Moltpellier 2, ENSCM 5253, cc 1501, Place Eugène Bataillon, 34095 Montpellier Cedex 5, France*
[d] *Dipartimento di Chimica, Fisica e Ambiente, Via del Cotonificio 108, 33100 Udine, Italy*

Contents

1 Effect of the exchange correlation functional **3**

2 Effect of basis set and hamiltonian **4**

3 ν_{CO} vs $\mathrm{CT}^{\pi}_{\mathrm{back}}$ **5**

1 Effect of the exchange correlation functional

We tested the effect of the exchange correlation functional by comparing the Charge Displacement functions (CDFs) obtained with the BLYP functional with the ones obtained with the LDA, BP86 and B3LYP functionals in $[Au(CO)]^+$ and [(Cl)Au(CO)] (S1). All functionals give very similar CDFs over the whole molecular space. In particular, BLYP, BP86 and LDA give essentially the same values for the DCD components of the Au-C bond whilst the B3LYP functional gives slightly different results. For $[Au(CO)]^+$, back-donation values are -0.18 (BLYP) and -0.15 (B3LYP) electrons and donation values are 0.34 (BLYP) and 0.30 (B3LYP) electrons. For [(Cl)Au(CO)], back-donation values are -0.33 (BLYP) and -0.28 (B3LYP) electrons and donation values are 0.23 (BLYP) and 0.20 (B3LYP) electrons. Remarkably enough, the total CDFs are almost independent by the functional used, since the differences on donation and back-donation compensate each other.

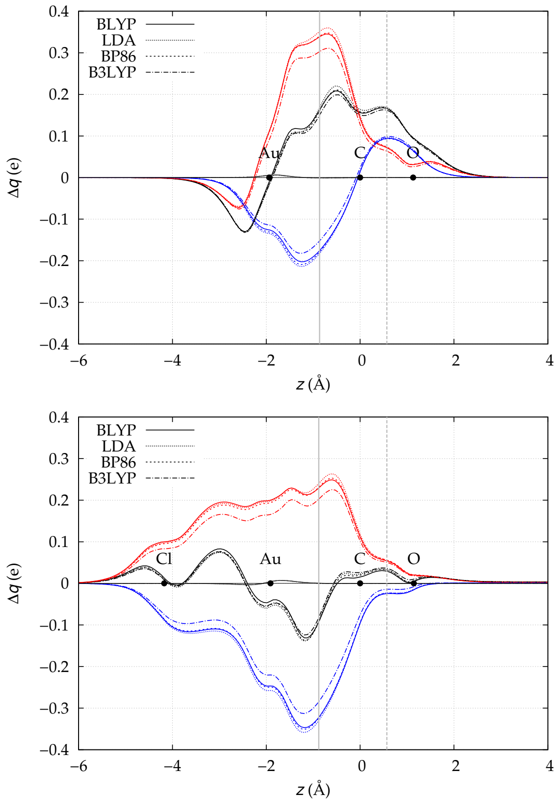

Figure S1 CDFs for the complexes $[Au(CO)]^+$ (top panel) and [(Cl)Au(CO)] (bottom panel). red and blue lines refer to the A_1 and B_1+B_2 components, respectively.

2 Effect of basis set and hamiltonian

The CDFs computed at the ZORA/TZ2P level are well converged with respect to the basis set. S2 shows that the ZORA/TZ2P CDF for both $[Au(CO)]^{+}$ and [(Cl)Au(CO)] is almost identical to that obtained with the limit basis set QZ4P. Moreover, both curves almost coincide with the CDF obtained by employing the

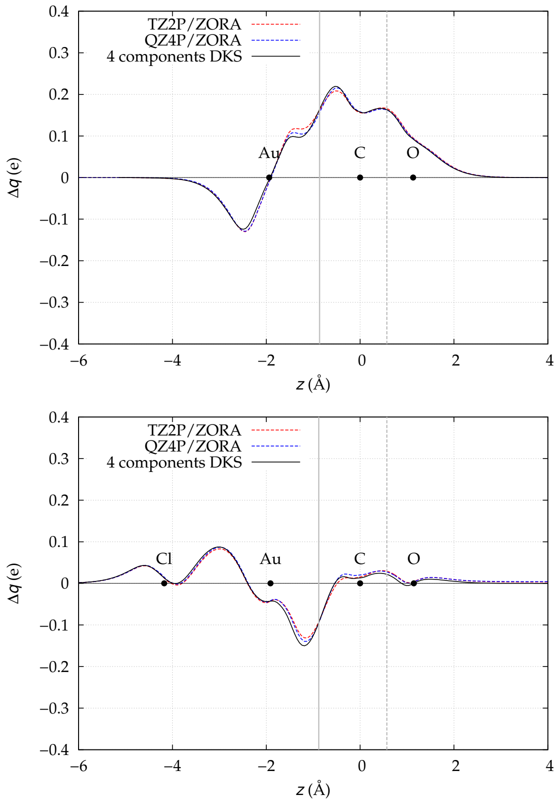

Figure S2 CDFs for the complexes $[Au(CO)]^{+}$ (top panel) and [(Cl)Au(CO)] (bottom panel). Solid black, dotted red and dotted blue lines refer to the net CD curve computed with the four component Dirac-Kohn-Sham (DKS) hamiltonian, BLYP/TZ2P/ZORA and BLYP/QZ4P/ZORA levels, respectively.

all-electron 4-component Dirac-Kohn-Sham (DKS) hamiltonian. We used the BLYP exchange-correlation functional in all cases. In the four-component calculation we used, for the large component, the basis set obtained by decontracting the Dyall basis set of triple zeta quality augmented with the related polarization and correlating functions (30s24p15d11f3g1h) on gold. The corresponding small component basis was generated using the restricted kinetic balance relation[1]. The calculation has been carried out with the DKS implementation of the molecular relativistic code BERTHA[2–4].

3 ν_{CO} vs CT^{π}_{back}

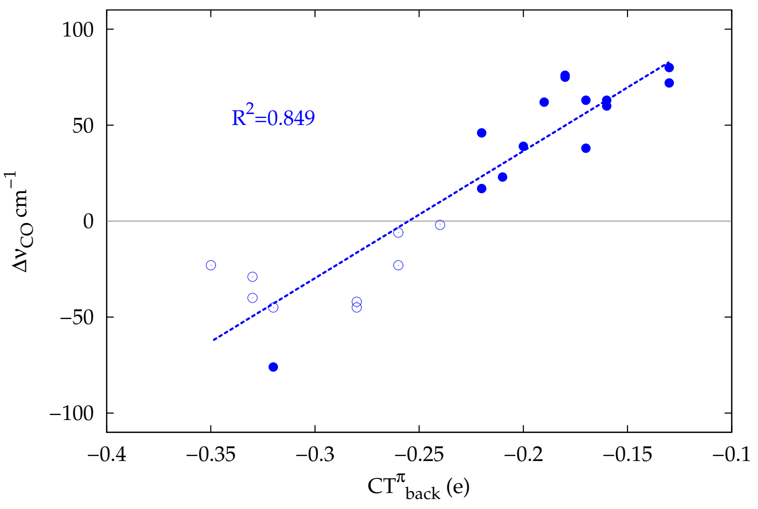

Figure S3 Correlation between the computed $\Delta\nu_{CO}$ in the considered series of $[(L)Au(CO)]^{0/+}$ complexes and the CT^{π}_{back}.

References

[1] Grant, I.; Quiney, H. *Physical Review A* **2000**, *62*, 022508.

[2] Rampino, S.; Belpassi, L.; Tarantelli, F.; Storchi, L. *Journal of Chemical Theory and Computation* **2014**, *10*, 3766–3776.

[3] Storchi, L.; Rampino, S.; Belpassi, L.; Tarantelli, F.; Quiney, H. M. *Journal of Chemical Theory and Computation* **2013**, *9*, 5356–5364.

[4] Belpassi, L.; Storchi, L.; Quiney, H. M.; Tarantelli, F. *Physical Chemistry Chemical Physics* **2011**, *13*, 12368–12394.
